# Evaluating flood dynamics and effects in Nagpur city using remote sensing and Shannon’s entropy analysis

**DOI:** 10.1038/s41598-025-86801-6

**Published:** 2025-02-10

**Authors:** Pranaya Diwate, Prasanna Lavhale, Chaitanya Baliram Pande, Saad Sh. Sammen, Samyah Salem Refadah, Mohd Yawar Ali Khan, Ismail Elkhrachy, Ali Salem

**Affiliations:** 1 Department of Geology, School of Basic and Applied Sciences, MGM University, Chhatrapati Sambhajinagar, Maharashtra India; 2 New Era and Development in Civil Engineering Research Group, Scientific Research Center, Al-Ayen University, Thi-Qar, Nasiriyah, 64001 Iraq; 3 Institute of Energy Infrastructure, Universiti Tenaga Nasional, 43000 Kajang, Malaysia; 4 Department of Civil Engineering, College of Engineering, Diyala University, Baqubah, Diyala Governorate 32001 Iraq; 5 Department of Geography and GIS, Faculty of Arts and Humanities, King Abdulaziz University, 21589 Jeddah, Saudi Arabia; 6 Department of Hydrogeology, Faculty of Earth Sciences, King Abdulaziz University, 21589 Jeddah, Saudi Arabia; 7 Civil Engineering Department, College of Engineering, Najran University, King Abdulaziz Road, 66454 Najran, Saudi Arabia; 8 Civil Engineering Department, Faculty of Engineering, Minia University, Minia, 61111 Egypt; 9 Structural Diagnostics and Analysis Research Group, Faculty of Engineering and Information Technology, University of Pécs, Pécs, Hungary

**Keywords:** Flash flood, Rainfall, Land use land cover, Shannon’s entropy, Geospatial analysis, Population, Hydrology, Natural hazards, Environmental impact

## Abstract

Flood is among the most disastrous natural disasters since they are responsible for massive damage to infrastructure, severe fatalities and injuries, innumerable economic losses, and social disruptions worldwide. These damages caused by floods have been worsening in recent years worldwide because of environmental degradation, climatic change, and high-speed urbanization. A rising precipitation rate increases the chances of floods in flood-vulnerable areas. A flash flood is a rapid flooding of geomorphic low-lying regions caused by remarkably high rainfall in a short duration. On September 23rd, 2023 a flooding event in the Nagpur, Maharashtra, it is directly impact on the human death and economic loss entire city. In the present study, the change in the dynamics of Nagpur city was analysed by employing remote sensing and GIS techniques to assess the change in the land use and land cover patterns. Landsat imagery of year 2000, 2010, 2020, and 2023 was used for land use and land cover classification. This analysis reveals that there is an increase in built-up area from 72.85 sq. km in year 2000 to 185.4 sq. km in year 2023. The built up land is increased this changes where directly affects the infiltration rate of rainwater into the soil. The total area covered by water bodies is reduced to 2.29 sq. km in 2023 which were 12.2 sq. km in year 2000. It is indicates the encroachment of built-up land on the water bodies. On the day of flash flood occurrence, it was observed that Nagpur city received 145 mm rainfall which is highest in the month of September, 2023. The Shannon entropy model was used to estimate the population dynamics and growth patterns of Nagpur city. Higher entropy values were obtained during the analysis which indicates the rapid transformation of city in all directions. Population dynamics of Nagpur city also indicate the inflation in population from 4,067,637 in 2000 to 4,653,570 in 2010. The SAR water index was calculated using Google Earth Engine to detect the water surges in residential areas during the flood. Precautionary measures should be taken by governing authorities to avoid such disasters. Proper city planning and improvements in drainage systems are recommended within the city. It is needed for an hour to develop a river monitoring system and early warning system, as well as preventive measures that should be implemented, like the construction of retaining walls to control the flood water.

## Introduction

A flood is a surplus of river water that spreads over a land area. Floods typically occur due to heavy rainfall events when watercourses cannot carry the excess water^1,2^. They can destroy entire areas, from the devastation of croplands and buildings to the ruining of the balance of the environment and the spreading of disease. The frequency and impact of extreme flood events have recently increased worldwide. All the regions with low elevations and populated territory of the tropical regions fall into flood risk-prone regions. Such areas are often flooded by both natural and anthropogenic agents^3–5^. Flood incidents are caused by heavy rain, cyclonic events, and by climatic variability. Hilly regions are least vulnerable to flooding events. The geological features of an area either directly, or indirectly, affect the infiltration and runoff rate based on the porosity and permeability of rocks and soil. Heavily populated areas have a higher chance of vulnerability to floods^6–9^. About 40% of natural disasters across the globe are caused by flooding due to changing in rainfall patterns^10,11^. It is important to note that human actions, such as improper water management and urbanization, significantly contribute to the severity and frequency of these floods. Rapid urbanization causes quick changes in the land use pattern of cities, which creates more impervious areas. It further increases runoff and overburdening the pre-existing drainage system^12,13^.

A flash flood is kind of riverine flood which occurs when a large quantity of water is discharged in a lesser time within a few minutes or hours after heavy precipitation. This is a common local phenomenon, and the short-term incident comes with little or no sign of warning^14,15^. The high growth rate of urban areas and the consequent loss of landscape results in high surface water runoff rates, which causes flash floods in the lower-lying regions even after moderate rainfall^16–18^.

The present study was carried out to analyse the flood dynamics of Nagpur city. Shannon Entropy model was utilised along with the remote sensing (RS) and Geographical Information System (GIS) techniques to understand the change detection and flood dynamics. Previous study that was carried out in this manner lacks the utilisation of advanced techniques and implementation of RS and GIS effectively. Arora et al.^19^ carried out study to understand the flood susceptibility of Middle Ganga Plain using Shannon Entropy Model as well as Islam et al.^20^ studied the Bagmati basin in Bihar following same methodology. But not similar study was carried out by any researcher on Nagpur region. Above mentioned literature also lacks the long-term analysis with respect to change detection and population dynamics. Sharma et al.^21^ studied flood susceptibility of Chandrapur district using Shannon’s Entropy Index Bivariate Statistical Models. Similar to this, advanced study of flood evaluation dynamics for Nagpur city was carried out and results were interpreted in this research paper. Land use land cover (LULC) analysis had been carried out of last 23 years with remote sensing techniques to study the changing pattern. Rainfall pattern as well as population dynamics was also studied to understand the effect of growth on all aspects. Google Earth Engine was used to calculate the SAR index which was not calculated previously by any author. It is recommended that, there is need of implementation of advanced techniques to monitor the flood and establishment of early warning system. Preventive measures such as construction of retention wall are also recommended to manage the flow of water.

### Flood events in India

India ranks second in the global ranking in terms of the number of persons killed by floods. As stated by the National Disaster Management Authority, nearly 40 Mha of land in India is vulnerable to flooding. According to Ministry of Jal Shakti reports, the most current estimate on flood-prone areas in the country is 49.15 Mha, out of which states like Odisha, West Bengal, Assam, Uttar Pradesh, and Bihar are largely impacted and other country also major issues related to flood (PIB, Aug 2022). Southwest monsoon also known as the Indian Summer Monsoon (ISM) season is the primary rainy season in India in which country receives over 80% to 85% of total annual rainfall. The cumulative rainfall over India from June to September has important implications for the socio-economic growth of the subcontinent. The increase in extreme rainfall events during ISM has been particularly strong in the last 50 years due to downpours and cloud bursting (NDMA Annual Report, 2022–2023). The Indian subcontinent experienced 649 disasters from 1915 to 2015. Among these, 302 disasters were due to floods, with an average of 3 floods annually. The loss of lives by floods changed from an average of 1000 per year in the 1965–1975 decade to 1700 per year in the 2005–2015 decade. The collective financial loss from 2005 to 2015 was about 2% of the current GDP of the India.

The mean of annual flood damage in the ten years from 1996 to 2005 was Rs. 4745 crores, while the comparable mean for the previous 53 years was Rs. 1805 crore (NDMA). A growing trend of urban flood disasters has been observed in the past few years, by which major cities have been largely impacted in India. Among them, the well-known flood events occurred in 2002 and 2003 in Delhi, Hyderabad in 2000, Mumbai in 2005, Ahmedabad in 2001, Kolkata in 2007, Chennai in 2004, Jamshedpur in 2008, Delhi in 2009, Surat in 2006 and Guwahati and Delhi in 2010 (NDMA). The most recent flood events in India, like the Kedarnath flash floods (2013), Brahmaputra floods (2012), Mumbai floods (2005), Chennai floods (2015), Leh flash floods (2011), Jhelum floods (2014), Kosi floods (2008), Kerala floods (2018) and the Ganga floods of Bihar (2019) are evidence of rising danger of hydrological disasters^22^. Major cities have experienced disruptions in transport, loss of life, property, power, and incidence of epidemics due to floods in India^23,24^.

### Flood mapping utilizing remote sensing and GIS

The conventional method of flood mapping depends on hydrological modelling. Ground surveys require more time are unfeasible in real-time situations, specifically in areas with rugged terrain and minimal accessibility, the numerical and machine learning modeling helpful to rainfall forecasting and climate change impact analysis. Remote Sensing gaining popularity for mapping of flooded areas, it is requiring less effort in data collection and less workforce^25–27^. The hydraulic and hydrological models combined with GIS and Remote Sensing to generate the flood plain and hazard maps proves helpful in flood susceptibility mapping^28–31^. The ML models also effectively are used for groundwater predication and river water level forecasting for understanding the flood risk level. The flood hazard map is produced by considering land use land cover patterns, the slope of the region, soil types, rainfall, drainage density, and distance from the main river^32–35^. Flood maps are beneficial for evaluating and managing disasters as it is showing the dynamics and extent of flooding^28,30,36,37^. In the last few years, emerging cloud-based technology like Google Earth Engine (GEE) has revolutionized the field of remote sensing and GIS. GEE has proved to be a powerful tool in flood mapping as it provides large-scale data with less effort and gives the desired output in less time. Generally, SAR imagery has been used for flood mapping as it would also provide data during cloud cover^38–41^. In addition to RS and GIS techniques, the Shannon entropy model can also be utilized to analyse urban sprawl as it is one of the most effective tools for analysis. It determines exactly how much information is present in a variable, establishing the groundwork for a theory based on the idea of information provided^6,42,43^. All map in this study were created with ArcGIS version 10.8 (https://www.arcgis.com/).

## Study area

**Fig. 1:**
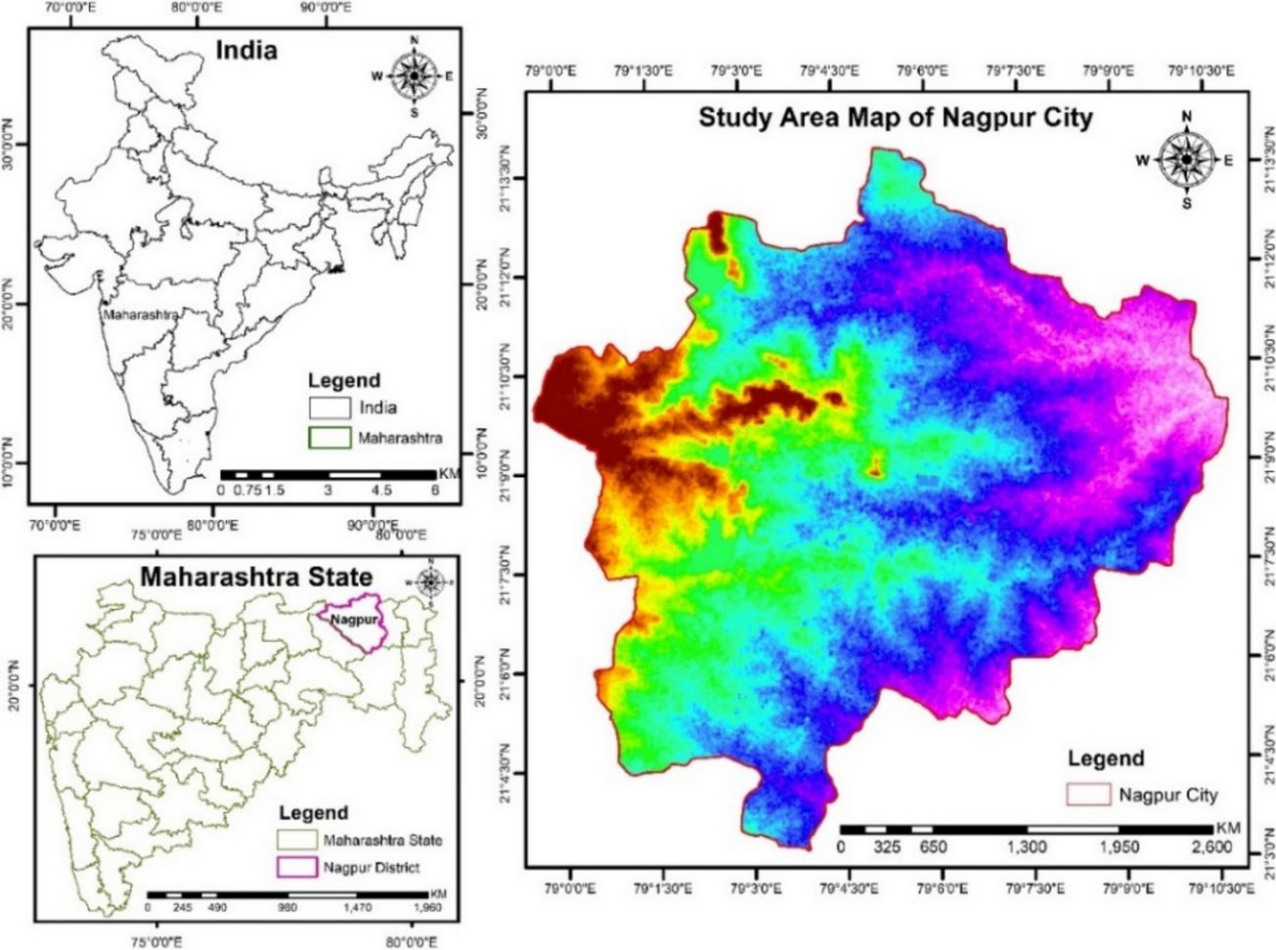
Study area map of Nagpur city.

Nagpur is situated on the eastward part of the Maharashtra state, the study area Latitude is 21° 9 N and 79° 6 E at an elevation of 303 m above mean sea level, spreading over an area of 217.56 km^2^ (Fig. 1). The city is marked by a denser population having approximately 11,000 persons per km^2^. Nagpur mainly has three rivers, namely Nag in the central part, Pili towards the Northern side, and part of the Pora River towards the Southern part^26^. This city that does not show vertical growth but shows horizontal radial growth^44^.

### Geology of study area

The Nagpur city and surrounding area have undulating topography spread over basaltic terrain, with Gondwana sediments beneath them. Geologically, the region is covered by basaltic lava flows of the Deccan trap, which belongs to the Upper Cretaceous to Eocene age. Sedimentary rocks are mostly seen in the northern portion of the Nagpur City having a succession of Kamthi and Talchir formations. The Kamthi formation comprises an overlapping layer of sandstone and shale. Talchir has a very limited area on the northward side and is composed of alternate layers of shale and sandstone exposed in a few places^44,45^. Chen et al.^46^ has studied on the stable and isotopes of rainfall on the global level and regional scale. The horizontal flows of basaltic lava spread over the majority of the western and southern parts of the city. Usually, these flows are divided by intertrappean beds. These formations range from a few meters to more than 200 m, and the size of each flow ranges from 11 to 24 m. The Alameda bed is a narrow belt in the city and the central and southeastern parts. These formations show the presence of limestone, clays, cherty limestone, and sandstone, with thicknesses ranging from 4 to 10 m. The primary rock types are schist, granite, pegmatite, granitic gneisses, etc., in the Northeastern part and Eastward region of the city^44^.

### Climate of study area

Nagpur has tropical wet and dry climatic conditions and usually shows high-intensity rainfall patterns. The mean precipitation is nearly 1161.5 mm annually. The winter is generally mild. The average temperature in the summer season usually increases from 28 °C and reaches to 35 °C. During the monsoon, the mean temperature decreases to 32 °C. On the other hand, the coldest period of the year in Nagpur particular in the month of December, when it has a 21 °C temperature on average but could lower to 12 °C^46^.

## Methodology

**Fig. 2:**
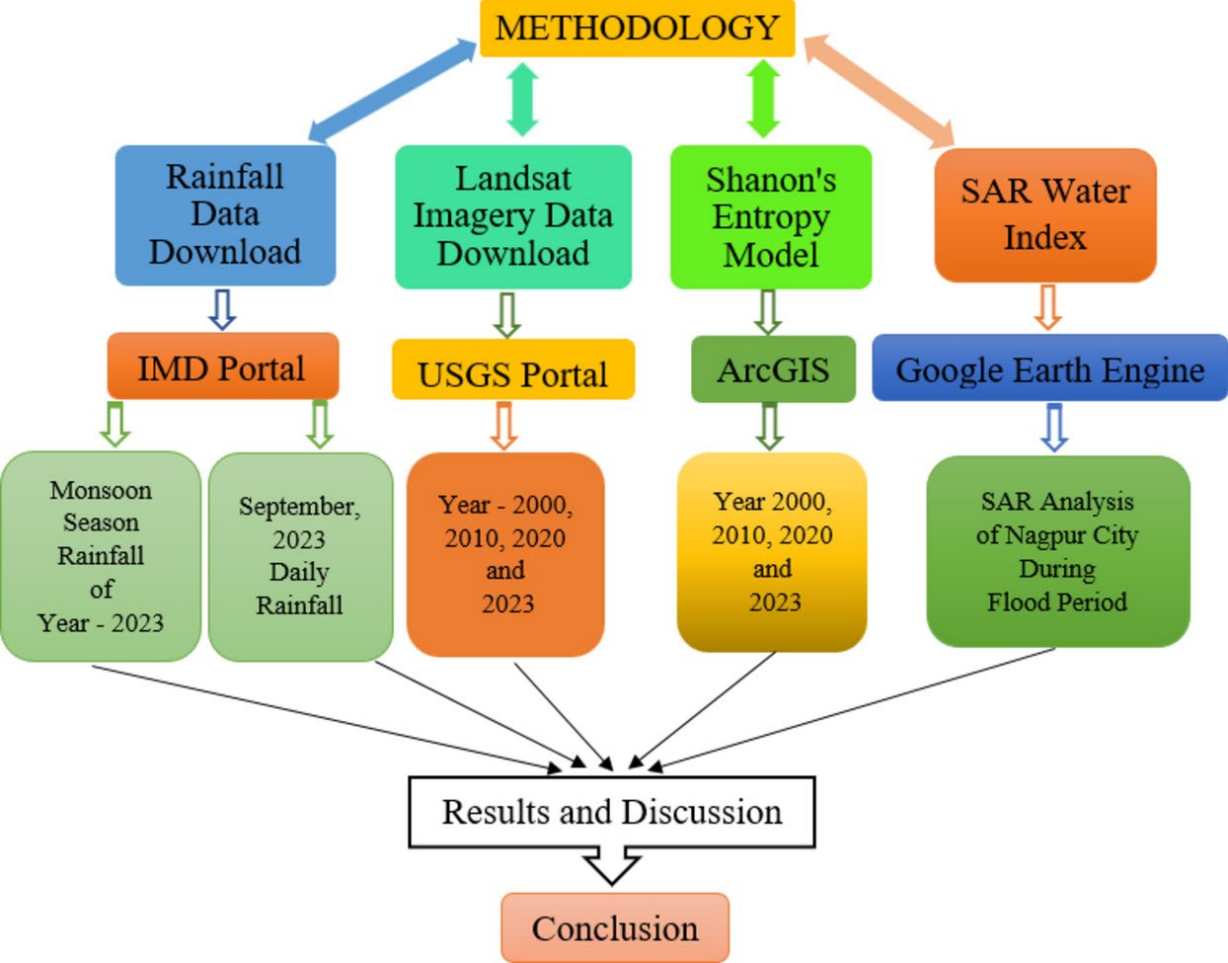
Flow chart of adopted methodology.

The flood mapping study for managing flood disasters, involves the use of various methods. These methods were employed to study the flood that occurred on 23rd September 2023 in Nagpur city (Fig. 2). The study included an examination of the rainfall variation over Nagpur city by comparing the data of 4 months. Analysis of land use land cover maps for year 2000, 2010, 2020, and 2023 to understand the variation was also carried out. The Shannon’s entropy model was implemented for a comprehensive examination of the change in Nagpur’s dynamics over time. Furthermore, Google Earth Engine was utilized to determine the SAR Water Index during the flood period in Nagpur.

### Rainfall data

The monsoon season rainfall data of Nagpur city was downloaded from the India Meteorological Department (IMD) portal. Specifically daily rainfall data for month of September 2023, during which flash flood occurred was downloaded and visualized through plotted graphs. These graphs effectively illustrate the daily variation in rainfall throughout the month. Additionally, the graphs of monthly rainfall data were prepared to demonstrate the seasonal variation over Nagpur city during the monsoon season.

### Land use land cover maps

Land use land cover maps of Nagpur city were made with the help of Landsat imagery. Landsat images of the year 2000, 2010, 2020, and 2023 were downloaded from the USGS Earth Explorer portal, among which Landsat-7 imagery was of the year 2000 and 2010 while Landsat-8 satellite imagery of the year 2020. Landsat-9 imagery data was downloaded for the year 2023. Landsat-7 imageries have a spatial resolution of 15 m for panchromatic bands, while Landsat-8 satellite imagery has a 15 m spatial resolution for panchromatic bands and a 30 m resolution for multispectral bands. On the other hand, Landsat-9 imagery has a resolution of 15 m, 30 m, and 100 m, depending on the spectral band. Date of sensing of imagery was chosen only 10% cloud cover datasets downloaded for selected this research investigation.

### Shannon entropy model

Shannon’s entropy calculates the quantity of information in a variable, which creates a foundation for a theory based on the concept of information. This method proves to be more precise and accurate in flood susceptibility modeling. Entropy measures system disorder, instability, uneven behavior, energy distribution, and uncertainty. It measures the consequences of judgments on different contested subjects and several anomalies between causes. The entropy index is the mean difference between unit group proportions from the whole system. Shannon entropy based on probability distribution and uncertainty assessment. The Shannon entropy model is built and runs to analyze the changes over time. Four maps of the year 2000, 2010. 2020 and 2023 were made and analyzed to understand the change in dynamics over the years.

### SAR water index

The SAR water index was calculated using the Google Earth Engine (GEE) platform. SAR satellite sensors possess unique ability to penetrate through clouds in all weather conditions. The sentinel-1 tools, capable of detecting open water bodies at high spatial resolution, are a crucial component. The total value across the VV and VH channels has been chosen to calculate low backscatter values in each channel, which indicate the presence of open water. The sentinel-1 satellites, with their dual-polarization capabilities (HH, VV, HH + HV and VV + VH) play a significant role in monitoring surface water. For the analysis of the SAR water index, SAR imagery of Nagpur city during the flood period from 22/09/2023 to 25/09/2023 was chosen.

## Results

The results obtained after the analysis are interpreted below. Precipitation analysis was carried out to know the variation in rainfall. Land use land cover analysis helps to understand the change in land use patterns within study area which was carried out using Landsat imagery. The Shannon entropy model play major role for change detection and provides results related to changes occurs over a period of time.

### Rainfall data analysis

**Fig. 3:**
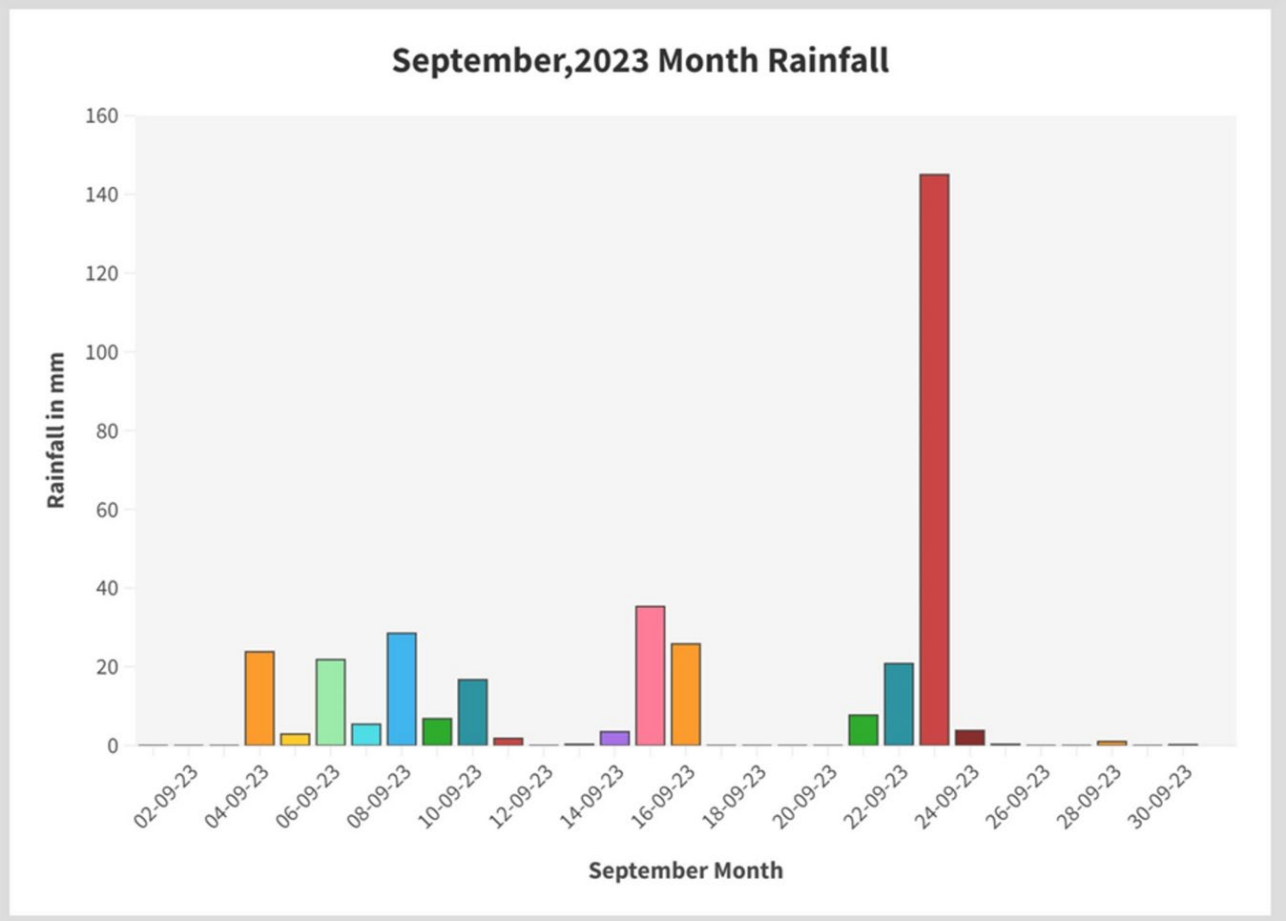
September, 2023 month rainfall over Nagpur city.

A graph of daily rainfall of month September, 2023 was plotted to understand the rainfall dynamics. It was observed that the actual rainfall for Nagpur city received in September, 2023 was 351.4 mm. The average rainfall observed is up to 35 mm. But on 23rd September 2023, Nagpur city received the highest rainfall i.e., 145 mm in a single day which resulted in flash flood (Fig. 3). The rate of precipitation is highly variable throughout September month ranging from 0 to 145 mm in a single day (Table 1).

**Table 1:** Daily rainfall data of September, 2023.

Date	Rainfall in mm
01-09-23	0
02-09-23	0
03-09-23	0
04-09-23	23.8
05-09-23	2.9
06-09-23	21.8
07-09-23	5.4
08-09-23	28.5
09-09-23	6.8
10-09-23	16.7
11-09-23	1.8
12-09-23	0
13-09-23	0.3
14-09-23	3.5
15-09-23	35.3
16-09-23	25.8
17-09-23	0
18-09-23	0
19-09-23	0
20-09-23	0
21-09-23	7.7
22-09-23	20.8
23-09-23	145
24-09-23	3.8
25-09-23	0.3
26-09-23	0
27-09-23	0
28-09-23	1
29-09-23	0
30-09-23	0.2

In July, 2023 Nagpur city received the highest rainfall of 491.2 mm followed by September, 2023 with 351.4 mm. The least precipitation of 122.9 mm was recorded in June, 2023 (Table 2). This data is crucial for understanding the variability of monsoon precipitation and will be instrumental in making more accurate future predictions.

**Table 2:** Monsoon season rainfall data for 2023.

Month	Actual rainfall
June	122.9
July	491.2
August	211.7
September	351.4

**Fig. 4:**
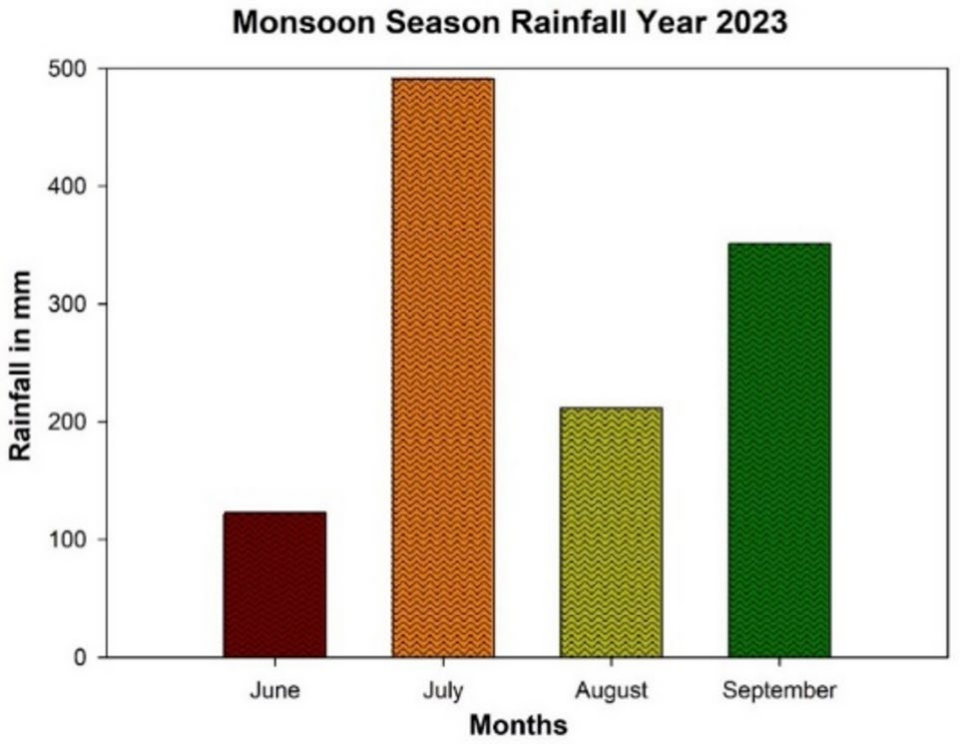
Monsoon season rainfall over Nagpur city of year 2023.

Minimum precipitation was observed during June, 2023 and a surge in rainfall was observed in July, 2023 which indicates frequent and heavy rainfall throughout the month. A precipitation decreases to 211.7 mm in August, 2023. Again, the September, 2023 higher precipitation was recorded. The graph shows the variability in intensity of rainfall throughout the monsoon season 2023 over Nagpur city (Fig. 4).

### Land use land cover variation analysis

The land use and land cover of area changes based on the demand for land for various purposes. The significant displacement of people from rural to urban areas over the past few decades has altered the dynamics of land use.

#### LULC analysis of year 2000

**Fig. 5:**
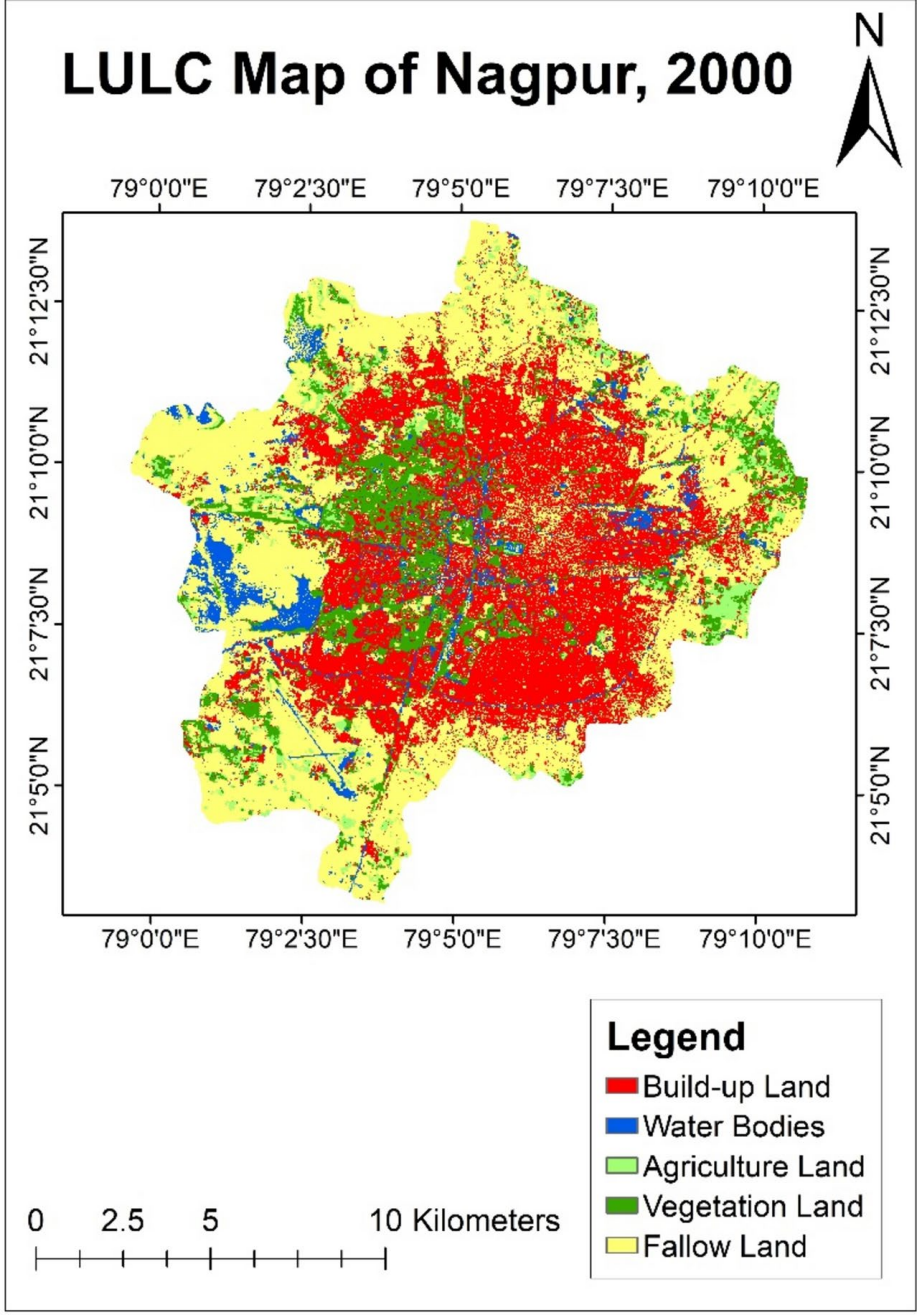
LULC map of Nagpur city of year—2000.

It was observed that in the year 2000, out of the total area of 211.73 sq. km of Nagpur city, a major portion was covered by fallow land covering an area of 89.4 sq. km (Table 3). Comparatively, the percentage of agricultural land and water bodies was only 5% of the total city area, covering 12.28 sq. km and 12.2 sq. km area of Nagpur city respectively. Vegetation was present in fewer parts of Nagpur city, having a 25 sq. km area. Presence of agricultural land was observed in the Eastern part of the city. Built-up land covers 72.85 sq. km of Nagpur city, which was 34% of the total area showing residential growth in the central part of the city (Fig. 5).

**Table 3:** Land use land cover distribution of year—2000.

Land classification	Area (sq. km)	Percentage
Build-up land	72.85	34.41
Water bodies	12.2	5.76
Agriculture land	12.28	5.80
Vegetation land	25	11.81
Fallow land	89.4	42.22
Total	211.73	100

#### LULC analysis of year 2010

In year 2010, a shrinkage of surface water bodies in Nagpur city was observed as their area drastically dropped to less than 1%. The city’s total area covered by water bodies was only 1.46 sq. km which were much less. On the other hand, the built-up area showed significant growth of 47.95% covering an area of 101.53 sq. km of Nagpur city (Table 4). This was the clear indication of urban expansion. Further, increase in population density was also observed during the years 2000–2010, which puts a stress on resources and emphasizing the need for sustainable urban development.

**Table 4:** Land use land cover distribution of year—2010.

Land classification	Area (sq. km)	Percentage
Build-up land	101.53	47.95
Water bodies	1.46	0.69
Agriculture land	3.8	1.79
Vegetation land	5.96	2.82
Fallow land	98.97	46.75
Total	211.72	100

**Fig. 6:**
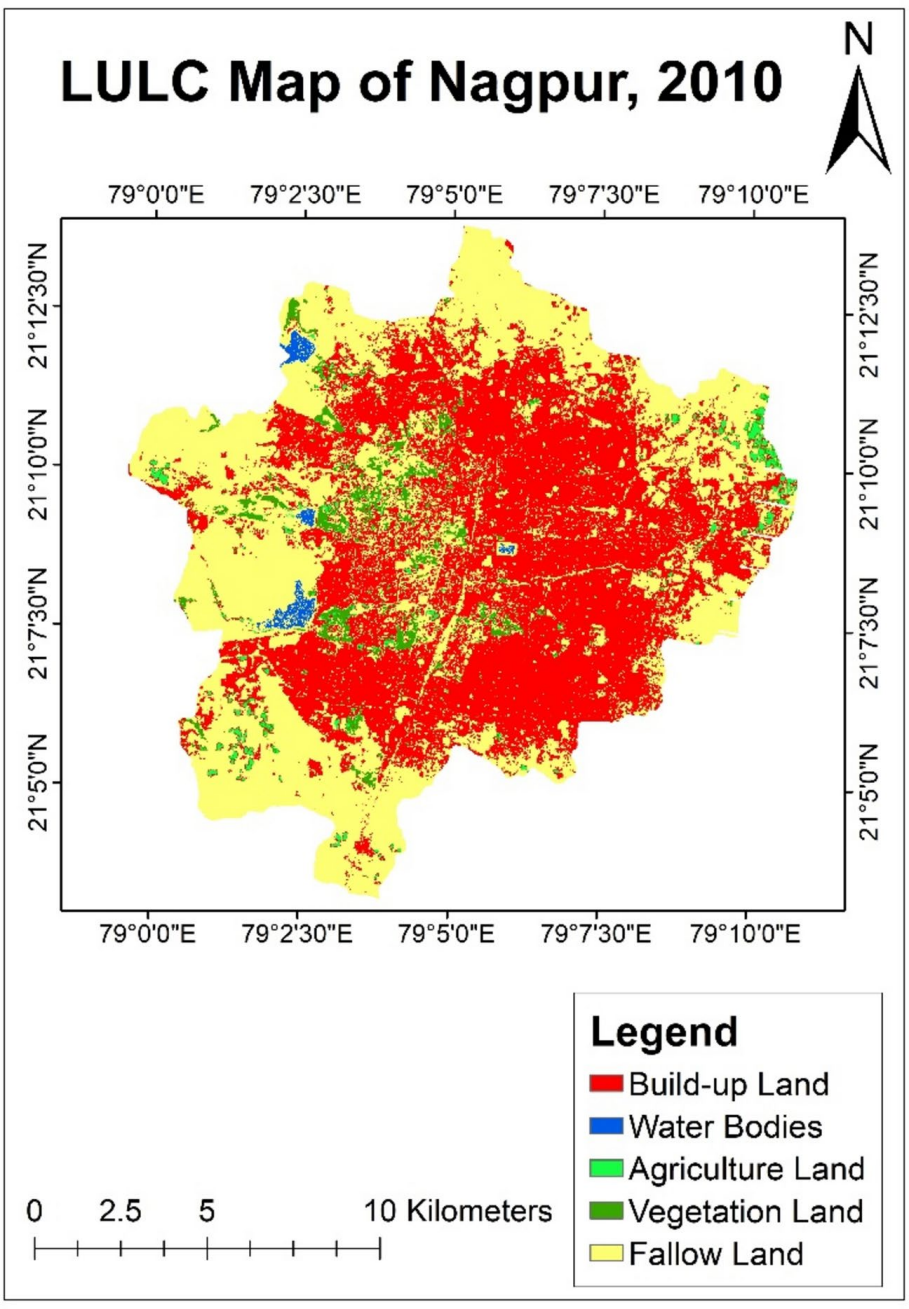
LULC map of Nagpur city of year—2010.

An increase in the percentage of residential area results in a decrease in the percentage of vegetative land and agricultural land within the city. Vegetative and agricultural land decreased to 5.96 sq. km and 3.8 sq. km respectively. The total percentage of the area covered by agricultural land was only 1.79%, and that of vegetation was 2.82% in the year 2010. It clearly indicated a reduction in vegetation within Nagpur city. This was caused due to the conversion of vegetative land to residential areas. On the other hand, the percentage of fallow land increased to 98.97 sq. km covering 46.47% of the total city area (Fig. 6).

#### LULC analysis of year 2020

During the year of 2010–2020, the built-up land in Nagpur city increased to 68.73%, covering a total area of 145.5 sq. km. This growth in urban development was a positive sign. However agricultural land had been critically reduced to a 0.18%, covering an area of 0.39 sq. km (Table 5).

**Table 5:** Land use land cover distribution of year—2020.

Land classification	Area (sq. km)	Percentage
Build-up land	145.51	68.73
Water bodies	2.29	1.08
Agriculture land	0.39	0.18
Vegetation land	6.11	2.89
Fallow land	57.41	27.12
Total	211.71	100

**Fig. 7:**
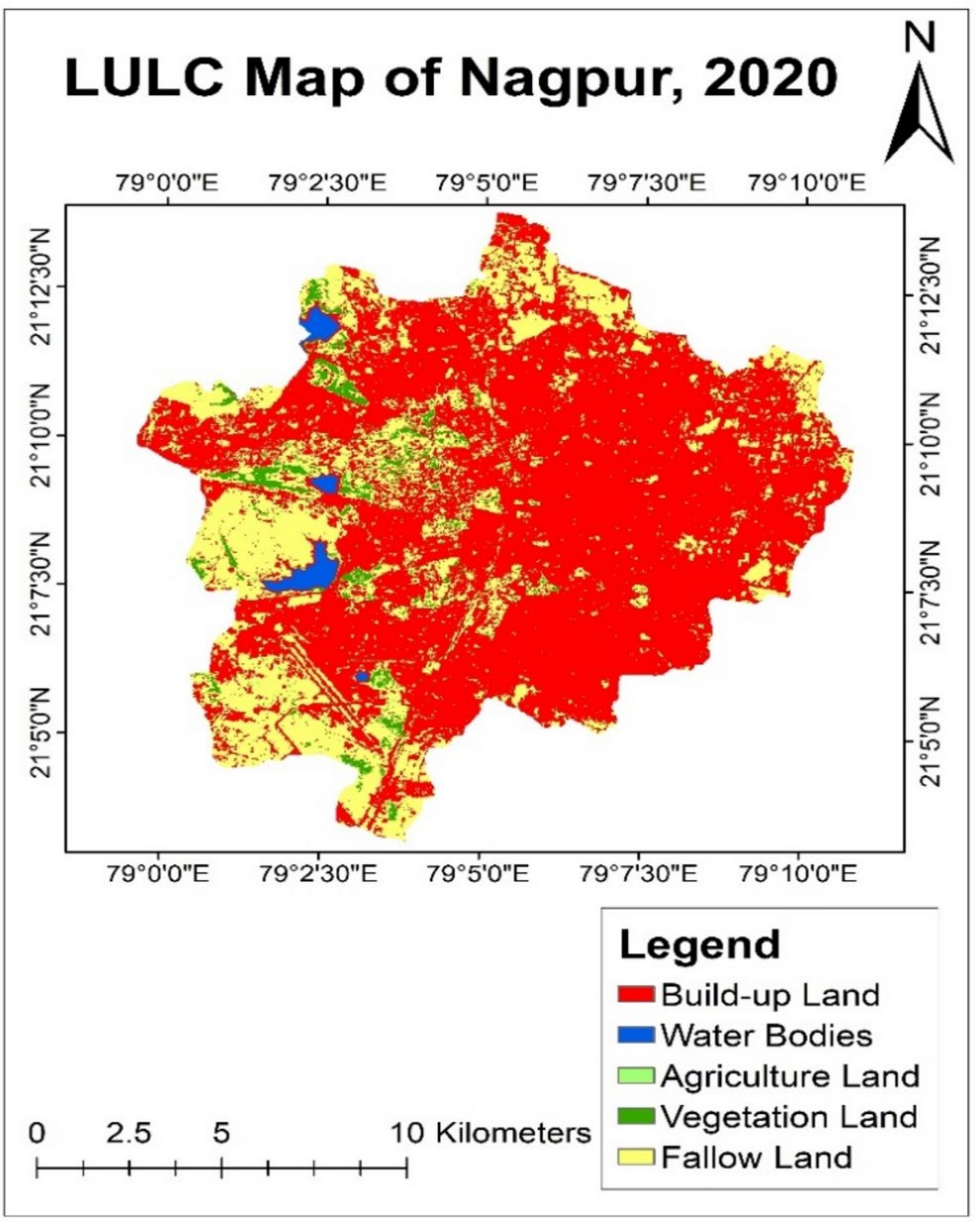
LULC map of Nagpur city of year—2020.

It was observed that the percentage of water bodies was increased above 1% which covers an area of 2.29 sq. km of total city area. It was observed that the rise in built-up land affects the fallow land and it gets reduced to 27.11% covering an area of 57.41 sq. km of total city area. The percentage of vegetation land increases to 2.88% having an area of 6.11 sq. km (Fig. 7).

#### LULC analysis of year 2023

During three years, from year 2020 to year 2023, the built-up land increased rapidly to 87.57%, covering an area of 185.4 sq. km (Table 6). The reason behind this was rapid urbanization, which required more residential space for a population. The area occupied by agricultural land was only 0.23% of city area, covering an area of 0.49 sq. km.

**Table 6:** Land use land cover distribution of year—2020.

Land classification	Area (sq. km)	Percentage
Build-up land	185.4	87.58
Water bodies	2.29	1.08
Agriculture land	0.49	0.23
Vegetation land	8.53	4.03
Fallow land	14.99	7.08
Total	211.7	100

**Fig. 8:**
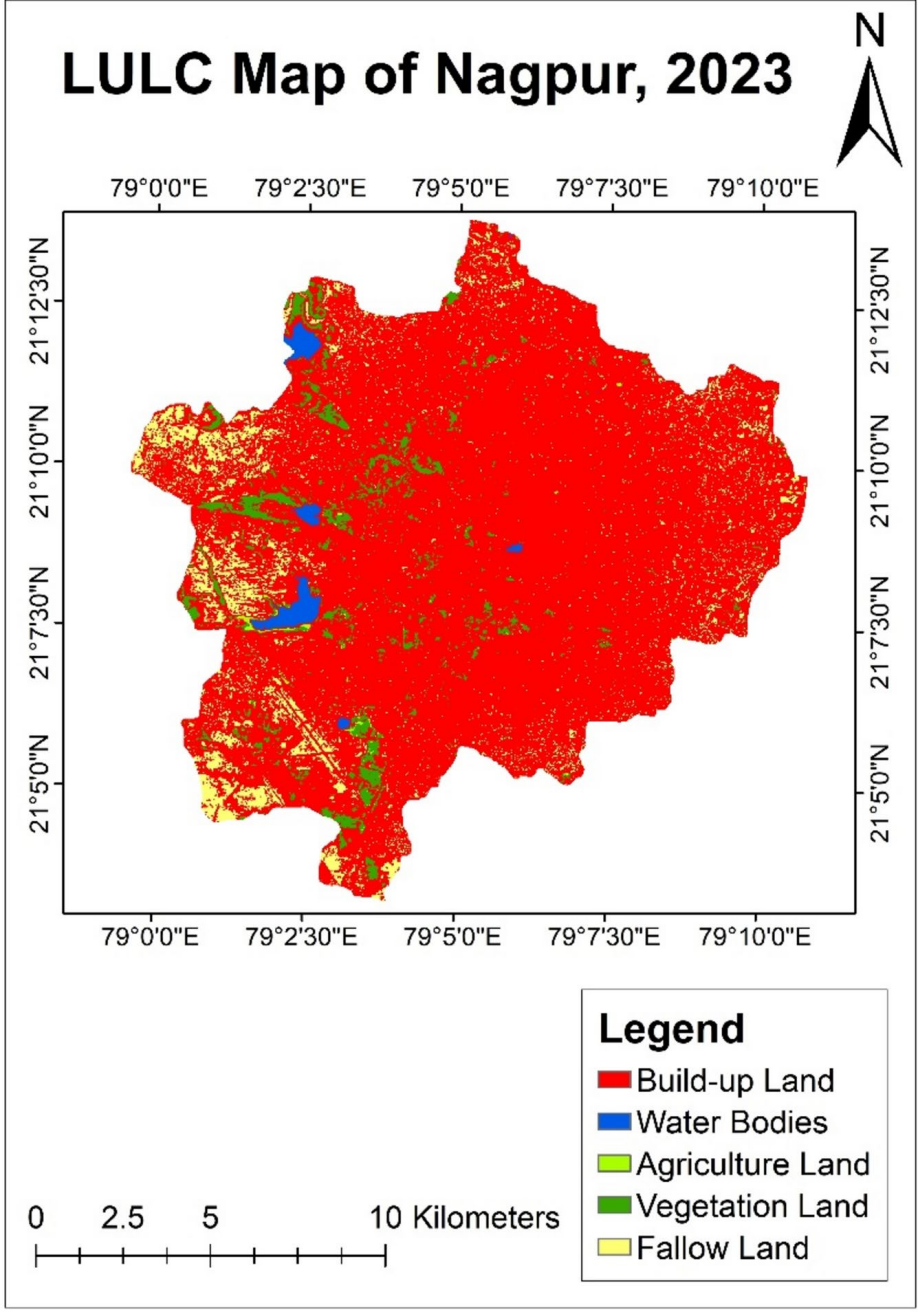
LULC map of Nagpur city of year—2023.

Water bodies cover an area of 2.29 sq. km having 1.08% of the total area. The growth in vegetation land was observed up to 4.02%, covering 8.53 sq. km. Fallow land further decreased to 14.99 sq. km which covered 7.08% of the total city area of Nagpur city (Fig. 8). It caused due to the conversion of fallow land to residential areas.

### Change detection analysis of Nagpur city

Change detection helps to analyse the changing dynamic of the city area Urbanization led to the conversion of fallow land and vegetative land to build-up land as an increase in population requires more space for living. This can be studied with the help of a change detection map, and interpreted results are discussed below.

#### Analysis of change in land use pattern during the year 2000–2010

**Fig. 9:**
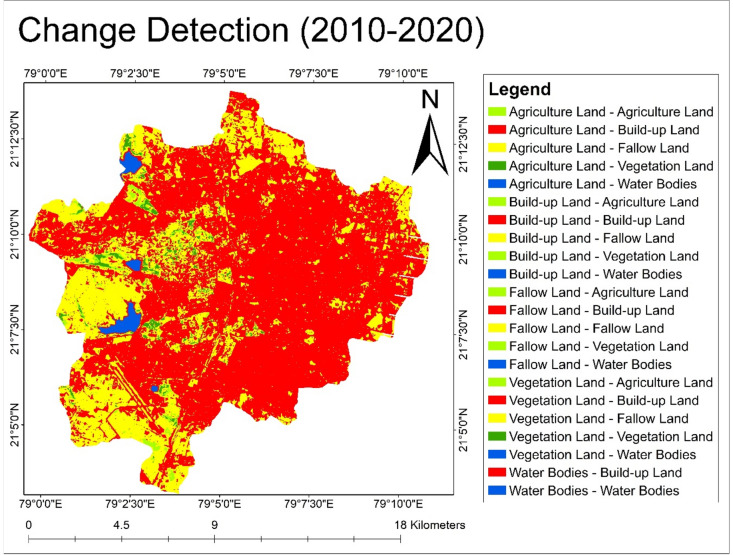
Change detection map of Nagpur city during year 2000–2010.

Major changes within Nagpur city were detected from year 2000 to year 2010. Agricultural land mainly observed on the Western side of the city (Fig. 9). It was observed that, conversion of 2.31 sq. km agricultural land to build up land for residential purposes led to decrease in vegetation within the city. Significant conversion of fallow land to build-up land of 23.50 sq. km was clearly visible in the Southwestern part of the city, which indicates rapid urbanization in this region. Nearly 7.33 sq. km of vegetative land was transformed into a build-up area. An increase in the buildup of land also led to the encroachment of city areas into the rural areas. It was noticed that 4.29 sq. km area of water bodies is transformed into a build-up area, which indicated an increase in the settlement around the water bodies.

#### Analysis of change in land use pattern during the year 2010–2020

**Fig. 10:**
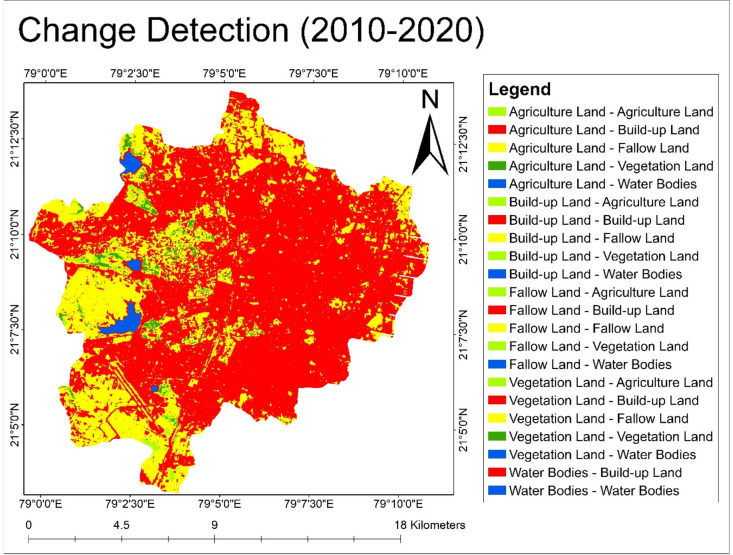
Change detection map of Nagpur city during the year 2010–2020.

During the period of ten years between year 2010 and year 2020, rapid urbanization was observed in all parts of Nagpur city (Fig. 10). About 48.76 sq. km of fallow land was transformed into the residential area during this period. The rapid increase in build-up areas over ten years decreased the percentage of fallow land and vegetative cover in Nagpur. Fallow land was observed in the western and southwestern parts of the city area. The overall growth of the build-up area also led to environmental degradation. Build-up expansion led to the encroachment of city areas into rural areas. An population increase then more demands of land for residential purposes which is gained by transforming existing fallow or vegetative land into residential areas. Agricultural land within the city only observed in the western part of the city in lesser proportion. Agricultural land of 1.30 sq. km and 0.58 sq. km of vegetation land was converted for residential purposes during this span. During this period, an increase in the area of surface water bodies was also detected. During the span of ten years, nearly 0.84 sq. km of fallow land had been transformed into water bodies.

#### Analysis of change in land use pattern during the year 2020–2023

**Fig. 11:**
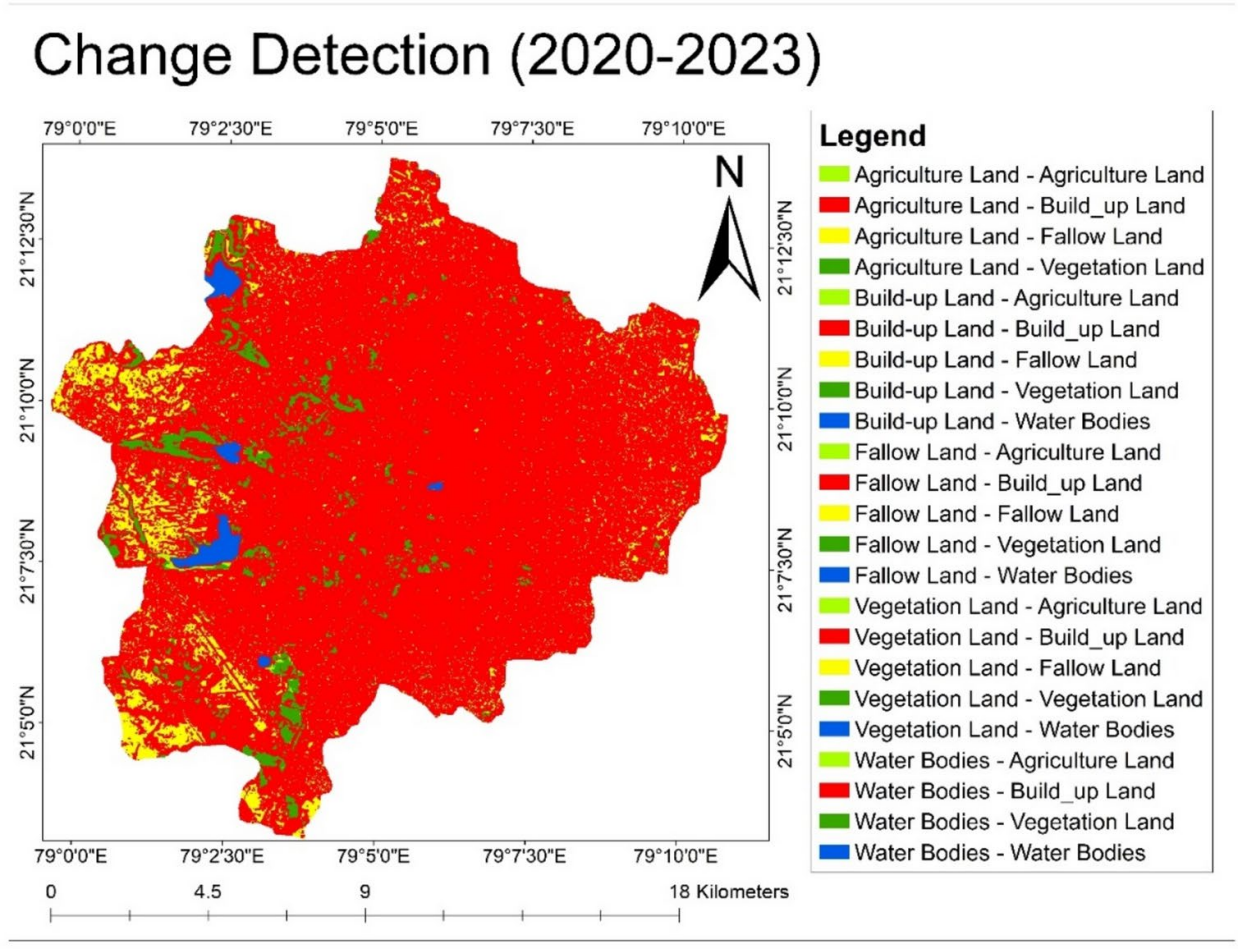
Change detection map of Nagpur city during year 2020–2023.

Build-up land was drastically increased within the span of three years, decreasing the area of fallow land. During the span of 3 years, 45.43 sq. km of fallow land was converted into build-up land. Increase in settlement around water bodies led to 0.13 sq. km area of water bodies transformed into residential areas. Agriculture land of 0.26 sq. km and vegetative land of 2 sq. km were transformed into build-up land, further decreasing the vegetative cover within Nagpur city (Fig. 11). A very less fallow land was observed in the western part of the city. The presence of agricultural and vegetative land was observed in lesser proportion. It was observed that the percentage of vegetative land rises to 4.12 sq. km as fallow land was transformed into vegetative land.

### Population dynamics analysis

**Fig. 12:**
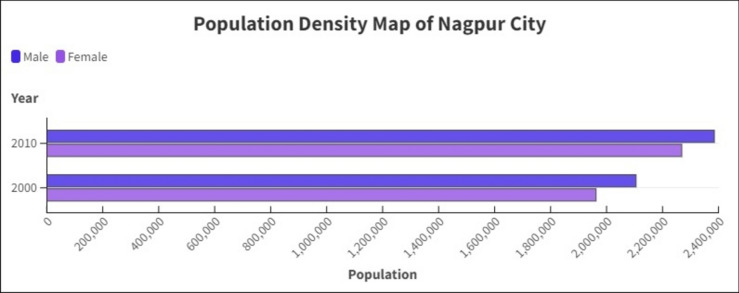
Population density map of Nagpur city.

Nagpur city demography changes over time as many peoples displaced from rural to urban area. It was observed that the population increased rapidly within ten years from year 2000 to year 2010 (Table 7). The rise in population directly impacts the city’s land use land cover patterns. From the available data, we can infer that the population of Nagpur city increased rapidly. It was also observed that, the male population has depleted by 0.75% in the year 2010, compared to the census of the year 2000. The female population shows hike in all these years (Fig. 12).

**Table 7:** Population data of Nagpur city.

	Year—2000	Percentage	Year—2010	Percentage
Total	4,067,637	100	4,653,570	100
Male	2,105,314	52	2,384,975	51.25
Female	1,962,323	48	2,268,595	48.75
Rural	1,453,886	36	1,474,811	32
Urban	2,613,751	64	3,178,759	68

It was observed that population of Nagpur city increases over the years. It caused due displacement of peoples from rural to urban areas. The rural population of the Nagpur area falls from 36% in year 2000 to 32% in year 2010. On the other hand, the urban population showed a hike of 4% from 64% in the year 2000 to 68% in year 2010. The increase in population changes the city dynamics, land use and land cover patterns and sometimes leads to encroachment in rural areas.

### Shannon entropy analysis

Shannon’s entropy study of Nagpur city helped to understand the changes occur within city during the year 2000 to year 2023. Many changes were clearly visible as pixel values showed variation over the years in land use patterns. Results obtained by after Shannon’s Entropy analysis are discussed below.

#### Analysis of Shannon’s entropy map of year 2000

**Fig. 13:**
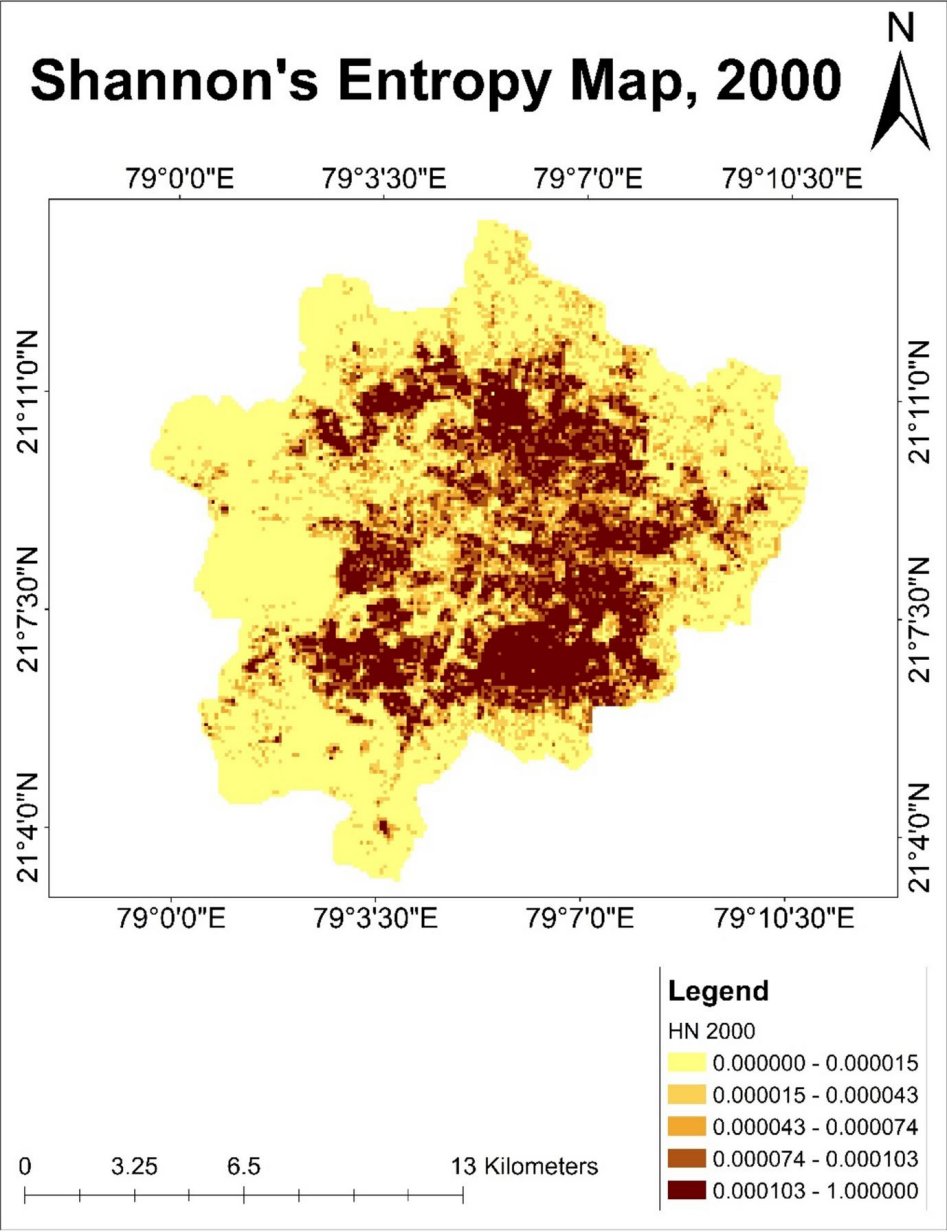
Shannon’s entropy map of Nagpur city for the year 2000.

Shannon entropy analysis of Nagpur’s urban growth indicated by the values ranging from 0 to 1. Light yellow to dark brown colour change illustrates the spatial distribution of entropy values across Nagpur. It indicated a change in the land use pattern of Nagpur city due to urban expansion. Shannon entropy maps showed the degree of randomness of land use patterns within the city in year 2000 (Fig. 13). Dark shades with higher pixel values showed the more excellent entropy value representing built-up and residential areas. At the same time, low entropy values indicated by light shade pixels indicates fallow land around the city area. Urban expansion not significantly influenced these parts of the city. It was observed that, urban development was concentrated in the central region and south-western part of the city area. Areas with low entropy values were the areas where urban development is less prevalent. On the other hand, higher entropy values showed urban growth near the water system, which pose a threat to the nearby area. It was observed that the map shows a heterogeneous spatial distribution of entropy values. It is essential to avoid urban growth near the water resources to prevent fatalities in the future.

#### Analysis of Shannon’s entropy map of year 2010

**Fig. 14:**
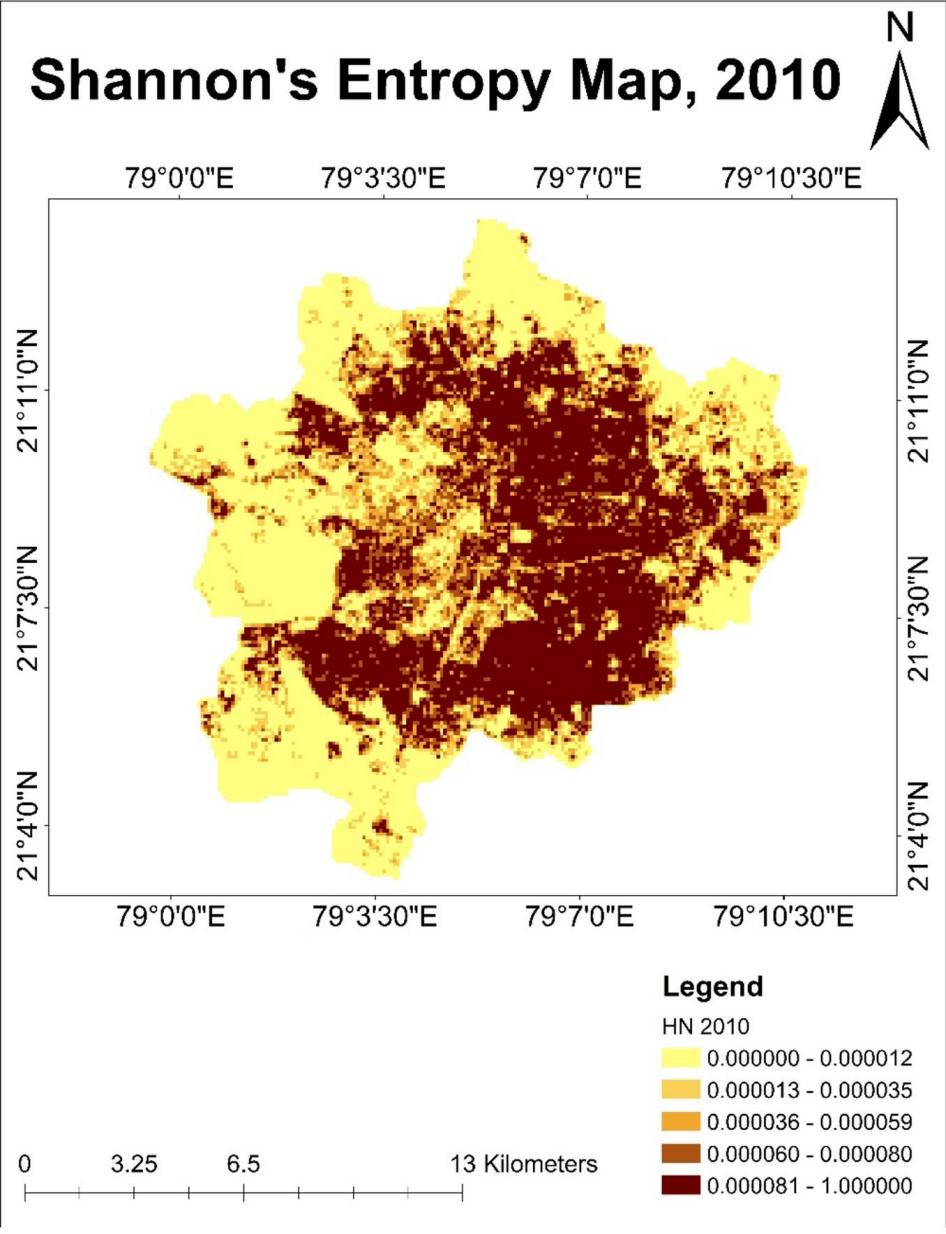
Shannon’s entropy map of Nagpur city for the year 2010.

Higher pixel values as compared to year 2000 was observed in year 2010. This means that urban areas expanded in all directions during this period. The presence of lower pixel values with lesser entropy values showed the absence of a built-up area on the outskirts of Nagpur city. This indicated a lesser percentage of urban expansion in this direction. Low entropy values suggest more uniform and predictable land use patterns. A single type of land use might dominate these areas. It was also detected that darker pixel values increase near the water bodies. It indicates that water bodies were surrounded by built-up areas. The higher entropy values seen in Nagpur’s central and south eastern parts indicate an increase in the built-up area around this corner of the city, which in turn leads to a higher level of disorder or unpredictability in land use patterns due to urban expansion (Fig. 14).

#### Analysis of Shannon’s entropy map for the year 2020

**Fig. 15:**
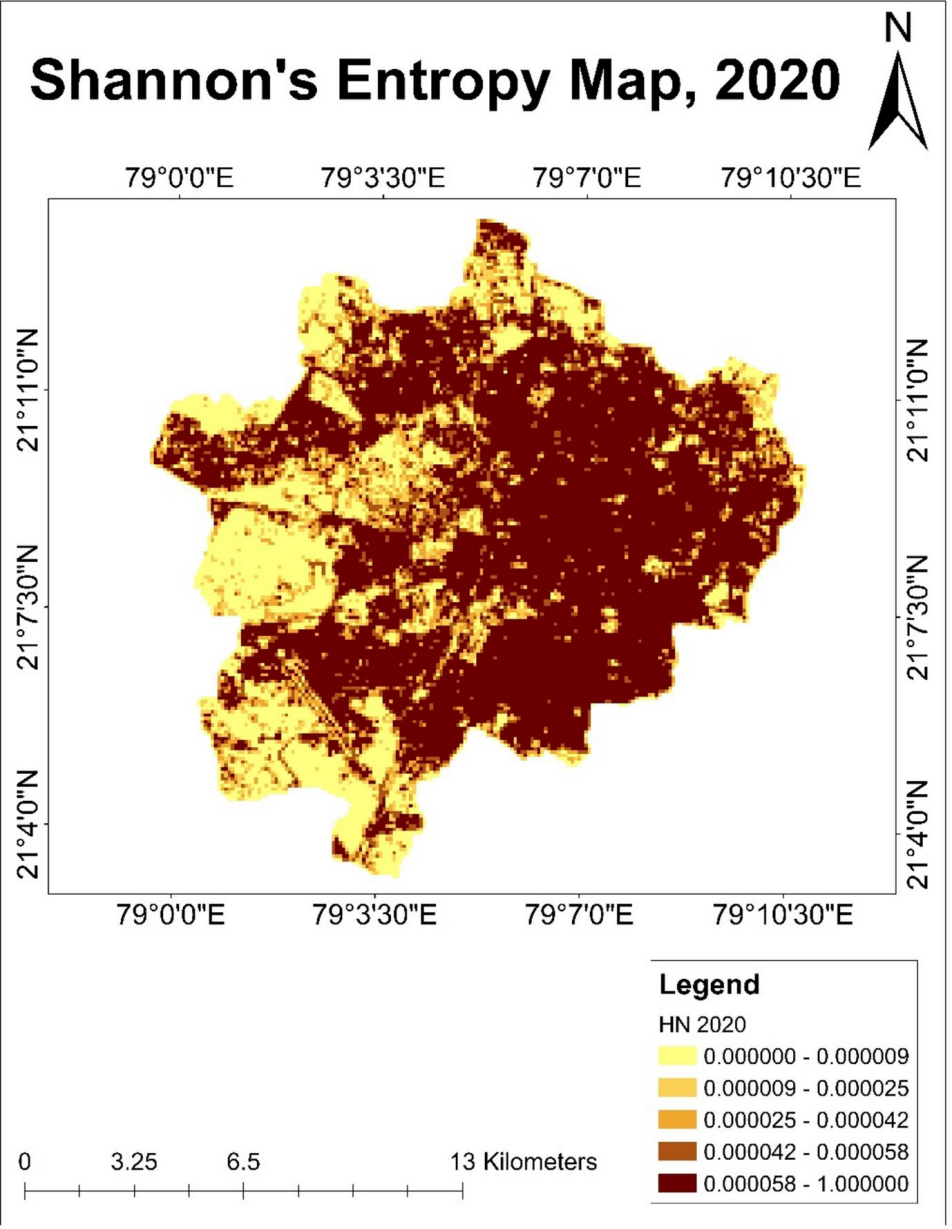
Shannon’s entropy map of Nagpur city for the year 2020.

It was observed that the, Shannon’s entropy map of the year 2020 indicates the rapid growth of urbanization in all directions of Nagpur city. Only the western and southwestern parts of Nagpur city showed lower pixel values which was indicated by the light colour. These areas were underdeveloped or used for agriculture or other non-urban purposes. Considerable encroachment of residential areas in the existing water bodies was also observed. Conservation of water bodies in city areas were essential as it plays a crucial role in balancing the ecosystem of surrounding areas. A more significant number of dark pixels which indicates a higher entropy value was observed in the eastern and south-eastern part of Nagpur city. It means this part of city experiences rapid growth of urbanization (Fig. 15). The central region and southwestern area of the region exhibit high entropy. This may be due to densely populated residential areas with complex and variable land uses. As compared to the year 2010, there was a decrease in pixel values which showed lower entropy values. A light pixel colour indicates the area with a lower entropy value. These are the areas that have the potential for further urban sprawl.

#### Analysis of Shannon’s entropy map of year 2023

**Fig. 16:**
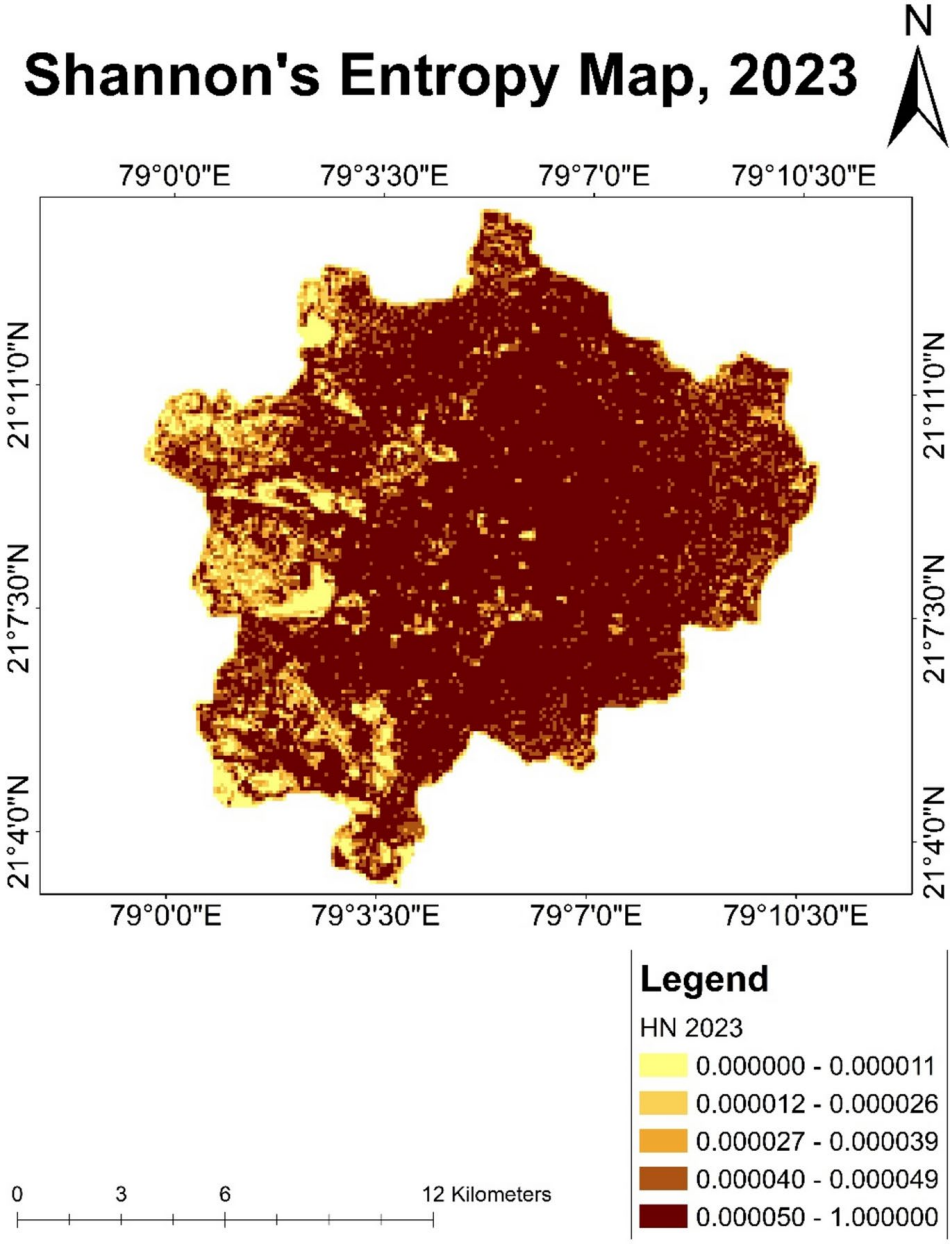
Shannon’s entropy map of Nagpur city for the year 2023.

It was observed that during the year 2020 to year 2023, Nagpur city experienced rapid urban development and most of the fallow land, agricultural land and vegetation land were converted to fulfil the increasing demand for land for residential purposes. High entropy values showed by the areas having mixed land uses reflecting significant urban development and expansion activities. Higher entropy values shown by darker pixels are present in nearly all parts of the city area except few areas in the western part of the city (Fig. 16). Lower entropy value was indicated by light colour, which was observed in lesser proportion in the western part of the city. These regions are either less developed or covered by agriculture or fallow land. High entropy zones were likely characterized by residential, commercial and industrial land uses suggesting dynamic and complex urban growth.

### SAR water index

**Fig. 17:**
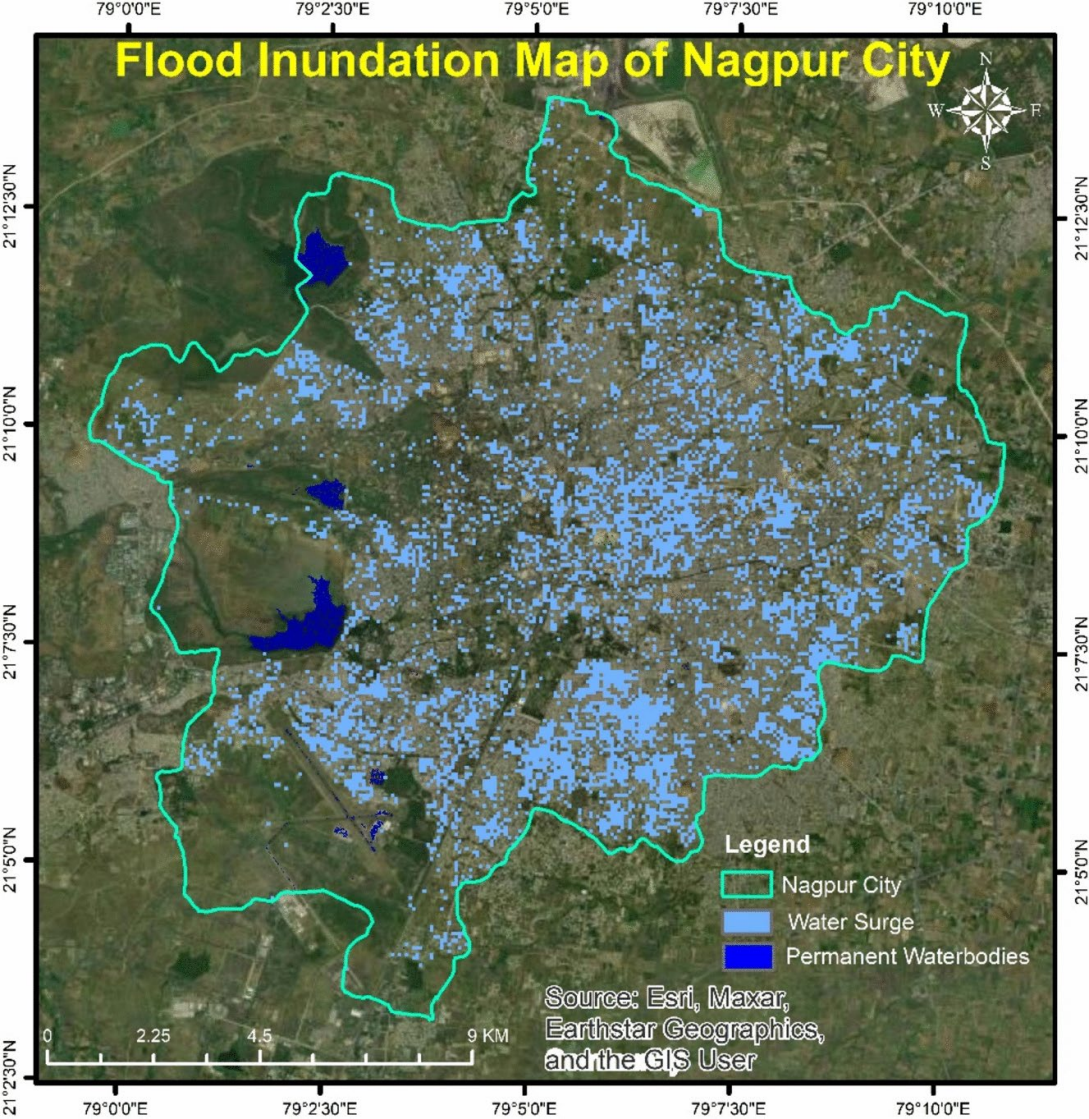
Water surge in Nagpur city during flood on 23rd Sept, 2023.

The SAR water index was calculated using Google Earth Engine. Sentinel-1 imagery during the flood period was used to visualize the water surge in the city area. Areas that were affected by the rise in water shown in Fig. 17. The SAR water index detected the presence of water in Nagpur city. Heavy rainfall in short duration causes flash floods, which affect residential areas. One of the reasons behind the spread of water in the city is the congestion in the drainage system. Water surges was observed all over Nagpur city. Central and southern areas of Nagpur city showed more accumulation of flood water. It indicates the areas with lower elevation and severely affected region. Denser urbanization was observed in the area, where higher water surges observed. Increases in residential areas also causes an increase in impervious structures, which results in higher runoff and accumulation of flood water. An increase in water level in residential areas causes damage to property and loss of lives.

## Discussion

Flood evaluation of Nagpur city reveals the factors responsible for the occurrence of flash flood. Heavy precipitation in lesser time and increase in built up land with poor management were the primary reasons behind this event. Variation in climate resulted in increase in precipitation intensity, and the urbanization led to the reduced imperviousness which was responsible for the flash floods. The eastward region of the Nagpur city has low-lying areas and agricultural land. The western areas are usually hilly and tablelands. Forest areas had been cut and urban utilities and residences have been built in the city. Nagpur city ranks as the third most populous city in Maharashtra state, having decadal growth of 19.21% during the decade of 2001–2011. This growth rate is greater than the state average for the urban population^24^. River monitoring and early monitoring system should be developed on priority basis to avoid such incident in future. It is essential to build retention wall for the management of flood water and drainage network should be properly maintained for the regulation of water.

## Conclusion

Rainfall plays a crucial role in flooding event. During the monsoon season of the year 2023, it was noticed that July month received the highest rainfall but the highest precipitation in a single day was observed on 23rd September, 2023 in Nagpur city which caused flash floods in the city. Along with geographical factors, many human-induced factors were responsible for the floods. The main component responsible for the flash flood was increase in the built-up area. The rapid population growth remarkably changed urban land use at different scales. Infrastructural development led to an increase in impervious structures. Such structure causes an increase in runoff after rainfall without water getting percolated in the soil. For better understanding, remote sensing analysis of Landsat imageries of year 2000, 2010, 2020, and 2023 was carried out of Nagpur city. Land use land cover mapping was done to analyse the changes over past 23 years after a regular interval. Land use patterns of Nagpur city indicate the conversion of agricultural land and fallow land to residential areas in large proportions. Encroachment of human settlement into the water body areas was also observed. An increase in residential areas near a water body poses a threat of flooding. An increase in the water level by any means resulted in the submergence of the nearby areas, which will cause loss of property and life. By studying the population dynamics of Nagpur city, it was noticed that there was an increase in population due to displacement of peoples from rural to urban areas. An increase in the urban population demands more land for residential purposes. Shannon’s entropy model was implemented to detect change in Nagpur city. After analysing the results given by Shannon’s entropy model, it can inferred that there were considerable changes in the dynamics of Nagpur. A higher entropy value shown by darker colour indicated a surge in built-up land. It was observed that the percentage of fallow land and agricultural land was decreased and comparatively residential areas were increased. Study of Shannon’s Entropy model also revealed that Nagpur city showed rapid development in the city’s central, eastern, and southwestern parts. On the other hand, the western part shows lesser entropy values which indicated the presence of agriculture land. Change detection maps help us to know the areas that have undergone tremendous transformation in a shorter period.

### Limitations

- During rainy season, it is challenging to capture data due to presence of clouds.
- Real time flood modelling requires availability of precise data so we have to rely only on SAR data.
- Limited accessibility during flooding makes it challenging to verify the accuracy of remote sensing data.
- Long revisit time of satellite can lead to miss important change occurred during a disaster.
- Unavailability of advanced system resulted in delay in data processing and analysis which can reduce the effectiveness of early warning system.

## Future aspects

- Drones can be used to gather the real time data during the disasters.
- Machine learning and AI models should be developed for the monitoring and prediction.
- Effective flood monitoring policy and infrastructure should be developed to reduce the flood risk.

## Recommendations

- Proper city and town planning is one of the key features to mitigate the occurrence of flood disasters. Construction of retention wall along the course of river is recommended.
- Regular maintenance of the drainage system and improvement in the sewer system is essential to avoid blockage due to trash.
- An early warning system and floodplain management system should be implemented in the watershed of the river to control the flood disaster.
- Rainwater harvesting can be done to reduce the runoff.

## Data Availability

The datasets used and/or analysed during the current study available from the corresponding author on reasonable request.
